# Editorial: C-Reactive Protein in Age-Related Disorders, Volume II

**DOI:** 10.3389/fimmu.2022.876431

**Published:** 2022-03-08

**Authors:** Mark Slevin, Blanca Molins

**Affiliations:** ^1^ School of Medicine and Pharmacy, George Emil Palade University of Medicine, Pharmacy, Science and Technology, Targu Mures, Romania; ^2^ Department of Life Sciences, The Regenerative Clinic, London, United Kingdom; ^3^ The School of Life Sciences, Manchester Metropolitan University, Manchester, United Kingdom; ^4^ Institut de Recerca Biomèdica August Pi i Sunyer (IDIBAPS), Barcelona, Spain

**Keywords:** monomeric C-reactive protein, cardiovascular disease, neuropathology, prognostic, diagnostic, therapeutic, cancer

In volume II of this Research Topic, we provide an update on the current research implicating C-reactive protein (CRP) in newly discovered disease pathobiology and providing up-to-date review articles summarizing novel potential therapeutic implications. Many of the leading researchers in the field of inflammation have contributed to this Research Topic and have focused on a variety of hot topics including the potential role of CRP in COVID-19 and prognostic implications where many studies have now shown it to be an independent determinator of severity of disease, cardiovascular complications and thrombosis, sepsis and ultimately mortality ( 1, 2 ). In this Research Topic, Ringel et al. provided a detailed case study of a 53-year-old COVID-19-positive patient with high circulating CRP levels, evidence of pneumonia, dyspnea and low oxygen saturation.

Following four successive days of CRP apheresis (with removal of around 79% of the protein), the patient showed almost a full recovery within the next 14 days. This data, for the first time, indicated a potential direct role of CRP in mediating or promoting the pathological processes associated with severe disease. A second paper in our Research Topic provided a critical review of the potential prognostic role of CRP in determining development and outcome in COVID-19 patients, and also an eloquent description of the mechanisms through which cellular and tissue binding of the protein (with concomitant dissociation into the monomeric form) could perpetuate the disease increasing its severity and ultimately mortality (Luan et al.).

The knowledge of the mechanisms and signaling pathways that CRP, and particularly the monomeric dissociated form mCRP, contributes to inflammation, are quite well established over recent years However, these are particularly important to understand, for example in diseases such as cardiovascular associated coronary artery atherosclerosis, where chronic low level inflammation involving raised blood circulating CRP (such as in autoimmune disease) could increase the risk of premature illness, risk of thrombosis and myocardial infarction. Novel additional concepts and capabilities are still being observed, for example, Siennicka ( 3 ), recently described evidence for a direct link between circulating dissociated-mCRP, platelet activation and aggregation and thrombogenesis, suggesting that microvesicle or platelet bound mCRP could be a novel marker of risk.

In the current Research Topic, Si et al. performed Mendelian randomization genome-wide-association studies using the UKs population-based biobank of 500,000 individuals to identify potential causal relationships between CRP, inflammation and disease. Correlations of phenotypic and single nucleotide polymorphic variants with conditions including hypercholesterolemia and neurological disease, provides further evidence and a platform for more detailed and focused analyses to be performed. Recognizing that CRP aggravates the innate inflammatory response partially through a complement-dependent mechanism, Zeller et al. significantly developed our understanding of the interplay between mCRP and white leukocyte blood cell subsets, by demonstrating specific reactive-oxygen species (ROS) generation only by classical monocytes and neutrophils in the presence of complement and not in the presence of native CRP. During their conclusions, they noted that ROS generation by leukocytes is a critical component in worsening sepsis, inducing endothelial dysfunction and tissue damage, and hence, blocking these pathways could be an important therapeutic approach.

To date, there have not been studies investigating the possible significance of CRP or mCRP distribution in cancer and any associated relationship with evolution or metastasis. Most of the previous data is eloquently summarized in a thorough review by Potempa et al. in this Research Topic and in reference to the groups previous findings and hypotheses. The findings of an overall reflection of circulating CRP levels predicting cancerous growth, prognosis and levels of tissue damage could be important in patient management, with rising levels being diagnostic and predictive of recurrence following remission; also within our Research Topic, O’Brian et al. describe the relationship between circulating CRP levels and pre and post-operative changes including IHC analysis of tissue samples in patients with renal cell carcinoma where higher levels of CRP were associated with tumor infiltration of T cells, and M2 macrophages correlating with increased mortality.

Previous studies from as long ago as the 1980s indicate potential tumoricidal effects of CRP in various cancer cell lines possibly through activation of the innate immune response of monocytes ( 4 ). Little work has described the potential effects of or analyzed by IHC mCRP within the tissue micro-environment of growing tumors, however, perhaps surprisingly, mCRP injection into murine models of breast adenocarcinoma significantly slowed down the growth of tumors compared with controls whilst native CRP had no effect; the mechanisms through which this inhibition occurs need further elucidation ( 5 ).

In summary, this Research Topic continues on from the original theme of CRP in aging and highlights new disease profiles recently cataloged and potential utilization in diagnostics and prognostics. The work presented in this Research Topic also demonstrates the early phase we are still in, regarding our understanding of the value and full recognition of the biological properties of CRP and how the protein interacts with cells and tissues within both the normal and pathobiological aging process.

## Author Contributions

Both authors contributed to the article and approved the submitted version.

## Conflict of Interest

The authors declare that the research was conducted in the absence of any commercial or financial relationships that could be construed as a potential conflict of interest.

## Publisher’s Note

All claims expressed in this article are solely those of the authors and do not necessarily represent those of their affiliated organizations, or those of the publisher, the editors and the reviewers. Any product that may be evaluated in this article, or claim that may be made by its manufacturer, is not guaranteed or endorsed by the publisher.
